# Three-dimensional mapping study of pure transverse acetabular fractures

**DOI:** 10.1186/s13018-022-03148-8

**Published:** 2022-05-13

**Authors:** Junran Li, Jingxiu Zhai, Yingchao Yin, Siyu Tian, Zhongzheng Wang, Ligeng Li, Zhiyong Hou, Yingze Zhang

**Affiliations:** 1 Department of Orthopaedic Surgery, Third Hospital of Hebei Medical University, Shijiazhuang, Hebei Province China; 2 Key Laboratory of Biomechanics of Hebei Province, Third Hospital of Hebei Medical University, Shijiazhuang, Hebei Province China; 3 Institute of Trauma Surgery, Second Hospital of Tangshan, Tangshan, Hebei Province China; 4 NHC Key Laboratory of Intelligent Orthopaedic Equipment, Shijiazhuang, Hebei Province China

**Keywords:** Transverse fracture, Fracture mapping, Heatmap, Computed tomography, AO/OTA 62-B1

## Abstract

**Background:**

To describe and analyze the morphological characteristics, location and frequency of pure transverse acetabular fracture lines through fracture mapping and quantitative measurements.

**Methods:**

Transverse fractures were retrospectively reviewed and analyzed. All computed tomography (CT) data were used for reconstruction and manual reduction. The reductive fracture fragments were graphically overlaid onto a three-dimensional (3D) right hemipelvis template. Then, the fracture lines were accurately depicted onto the surface of the 3D template. The fracture lines were overlapped onto the model to create the 3D fracture map and heatmap. All cases were subdivided into infratectal (62-B1.1), juxtatectal (62-B1.2), and transtectal (62-B1.3) types based on the AO Foundation/Orthopedic Trauma Association (AO/OTA) classification. Some anatomic parameters of the transverse fractures were also analyzed in these 3 groups.

**Results:**

Our study included forty-nine transverse fractures from 32 male and 17 female patients (mean age, 42 years; range 21–74 years) and included 19 type 62-B1.1, 17 type 62-B1.2, and 13 type 62-B1.3 fractures. The average anterior rim fracture angle was 70.0° (± 11.6°), and the posterior rim fracture angle was 92.4° (± 28.5°). The anterior rim fracture angles in 40 cases (40/49, 81.6%) fell within a wide range between 63° and 80°. On the heatmap, the hot zones were located on the highest position of the cotyloid fossa and the narrowed region, and the cold zone was on the inferior third of the articular surface. For type 62-B1.3 fractures, the hot zone was located on the posterior of the acetabular dome. There were no significant differences in anterior rim fracture angle and anterior height among the three patterns (*P* = 0.071, *P* = 0.072). Post hoc tests of the posterior rim fracture angle and the posterior height revealed significant differences among fracture subtypes (*P* < 0.01). The posterior intra-articular fracture line was significantly longer than the anterior intra-articular fracture line in type 62-B1.1 and type 62-B1.2 fractures (*P* < 0.01).

**Conclusion:**

The fracture lines of transverse fractures through the anterior rim were concentrated on the narrowed zone, and the posterior fracture lines were diffusely distributed. The intra-articular fracture line distribution was focused on the superior and middle thirds of the joint surface. The recurrent fracture lines involving the weight-bearing dome mainly converged on the posterior region of the roof.

## Introduction

Pure transverse acetabular fracture is defined as an elementary fracture type in the Judet–Letournel classification because of the simple geometric form of its fracture line [1, 2]. However, in clinical practice, surgical treatment may be difficult considering that lesions of this type involve both anterior and posterior columns [3]. Moreover, most patients have concomitant injuries of the acetabulum and pelvic ring [4]. These conditions result in large challenges in the surgical treatment of transverse acetabular fractures [5]. To date, the incidence of postoperative complications still remains high [6].

The Judet–Letournel, Marvin Tile, AO Foundation/Orthopedic Trauma Association (AO/OTA), and 3-column classification systems for acetabular fractures have been used to describe fracture patterns and provide treatment protocols [7–10]. The traditional Judet–Letournel and latest AO/OTA classifications are common clinical classification methods that have categorized pure transverse acetabular fractures into infratectal (62-B1.1), juxtatectal (62-B1.2), and transtectal (62-B1.3) types according to the level at which the fracture ruptured the acetabulum [9, 10]. These three kinds of subtypes also indicate whether the roof was involved, which could help in surgical planning and prognosis prediction. Although orthopedists have devoted considerable attention to the fracture line locations on the articular surface, it might be quite difficult to grasp preoperatively whether the acetabular dome was broken by using conventional radiographic imaging. Computed tomography (CT) is appropriate for obtaining an accurate assessment of acetabular fractures; even so, the configuration of the fracture line may still be unclear visually. In addition, the shapes of the transverse fracture lines can be relatively diverse. However, the above-mentioned factors are essential for making a preoperative plan and achieving an excellent clinical outcome [11]. The fracture mapping technique created by Armitage et al. based on three-dimensional (3D) CT has been widely utilized to elucidate the patterns of fracture lines, especially in irregular bone and intra-articular fractures [12–14]. With increasing concern over precise treatment and the application of various medical image postprocessing software, fracture mapping has been increasingly more common in orthopedic fields. However, to the best of our knowledge, this approach has rarely been used to analyze the distribution of acetabular fracture lines, such as models of transverse fractures.

The purpose of this study was to describe and analyze the morphological characteristics, location and frequency of pure transverse acetabular fracture lines through fracture mapping and quantitative measurements. We hypothesized that our study findings could reveal classic fracture lines and recurrent fracture zones that might provide better understanding of the features of pure transverse acetabular fracture and theoretical guidance for preoperative surgical planning.

## Methods

### Subjects

This retrospective study included patients with acetabular fractures from January 2014 through September 2021 and was approved by the required institutional review boards of two level-I trauma centers. All cases were diagnosed and categorized according to the Judet–Letournel and AO/OTA classification systems using radiographs, CT scans, and surgical reports by two trained orthopedists. The inclusion criteria were pure transverse acetabular fracture, sufficient CT image quality, and a slice thickness of ≤ 1 mm. The exclusion criteria consisted of an age younger than 18 years or CT image that did not show a completely affected pelvis. A total of 49 pure transverse acetabular fractures in 49 patients fit the inclusion and exclusion criteria and were included in this study. All cases were also classified into subtypes using the AO/OTA classification.

### Fracture mapping

**Fig. 1:**
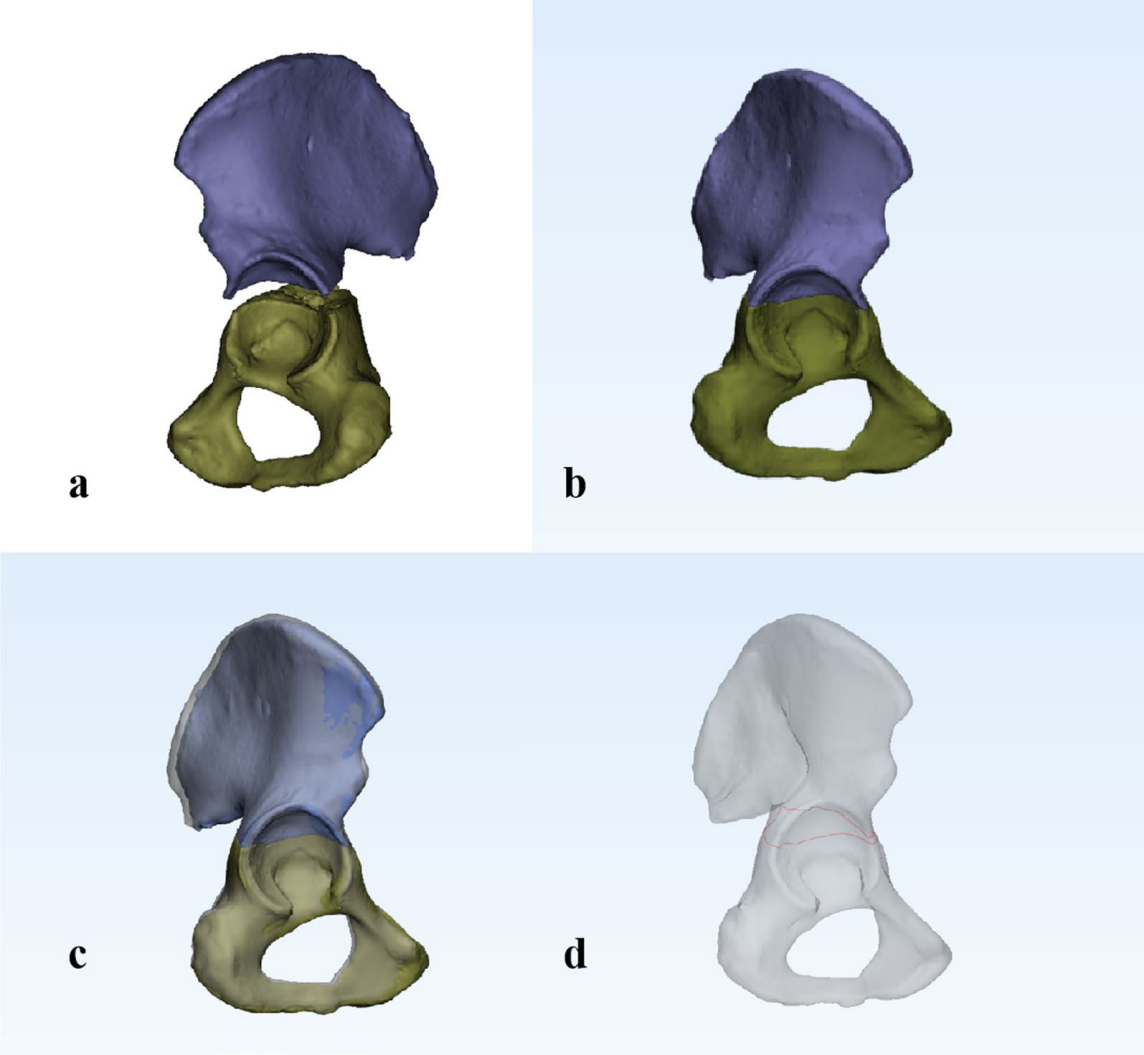
The method of creating a map of transverse acetabular fractures. **a** Fragments of affected hemipelvis were retained and separated into two individual parts in Mimics. **b** Reconstructed fracture fragments of the left-sided pelvis were reduced and mirrored to match the orientation of the right-sided standard model in 3-Matic. **c** The affected hemipelvis was moved and rotated to best match the standard model of the acetabulum and superimposed on the standard model at the same position. **d** The fracture line was drawn on the 3D template

The Digital Imaging and Communications in Medicine (DICOM) files of CT scans were loaded into the Mimics 20.0 medical imaging program (Materialise, Leuven, Belgium) to create a 3D reconstruction of the affected hemipelvis. Fracture lines were drawn according to the mapping method described by Yin et al. [15] and were used in this study. After 3D reconstruction, the affected hemipelvis was retained and separated into two fracture parts. Virtual and manual anatomical reduction was performed, and separated fracture fragments were merged into an original innominate bone with a single fracture line. An intact right hemipelvis was individually reconstructed from a healthy adult and served as the standard 3D model. Then, the reductive hemipelvis and 3D template were copied and exported into 3-Matic 12.0 (Materialise, Leuven, Belgium) software. If the left side of the acetabulum was affected, the mirror image function in 3-Matic was used to fit the right-sided standard model. The appropriate transparency of the 3D standard model was established, and reductive hemipelvis was superimposed on the normalized model using manual maneuvering. To align the two hemipelvises precisely in the same position, both the affected hemipelvises was scaled into a similar size as the standard model and the main anatomic landmarks were matched congruently. The acetabular fracture lines were drawn and overlapped accurately onto the surface of the model using smooth curves in 3D view (Fig. 1). Ultimately, heatmaps [16, 17] based on the fracture lines were generated after transferring the data to E3D software (Central South University, Changsha, China).

### Anatomic parameter assessment

**Fig. 2:**
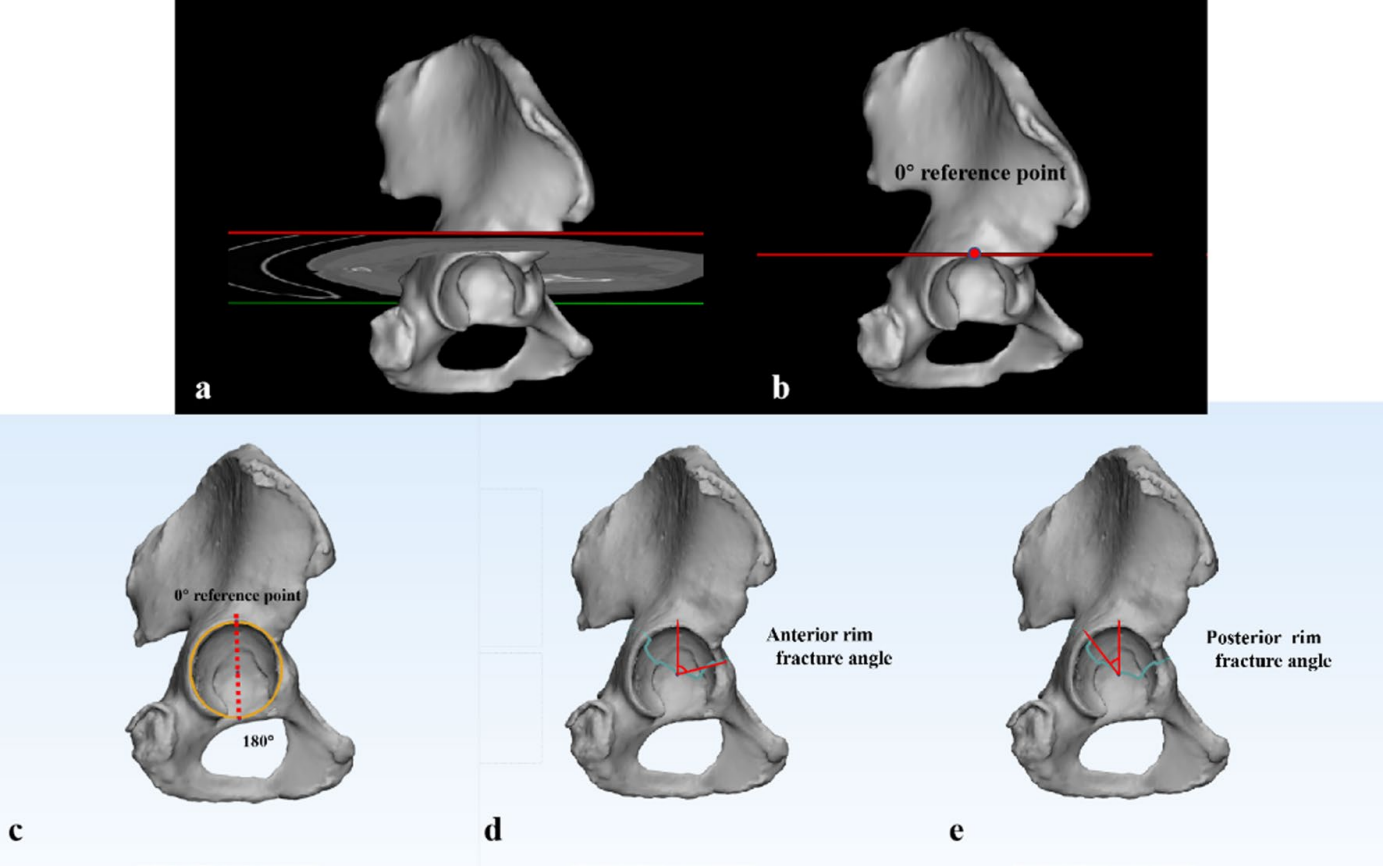
**a** Three-dimensional reconstruction of a hemipelvis on an axial CT cross section through the highest position of the acetabulum. **b** The tangent point on the acetabular roof is regarded as the 0° reference point. **c** Illustration of a clock face over the acetabular rim. **d**, **e** The anterior or posterior rim fracture angle was measured as the intersection between the 0° reference point and the fracture position on the anterior or posterior rim, respectively

**Fig. 3:**
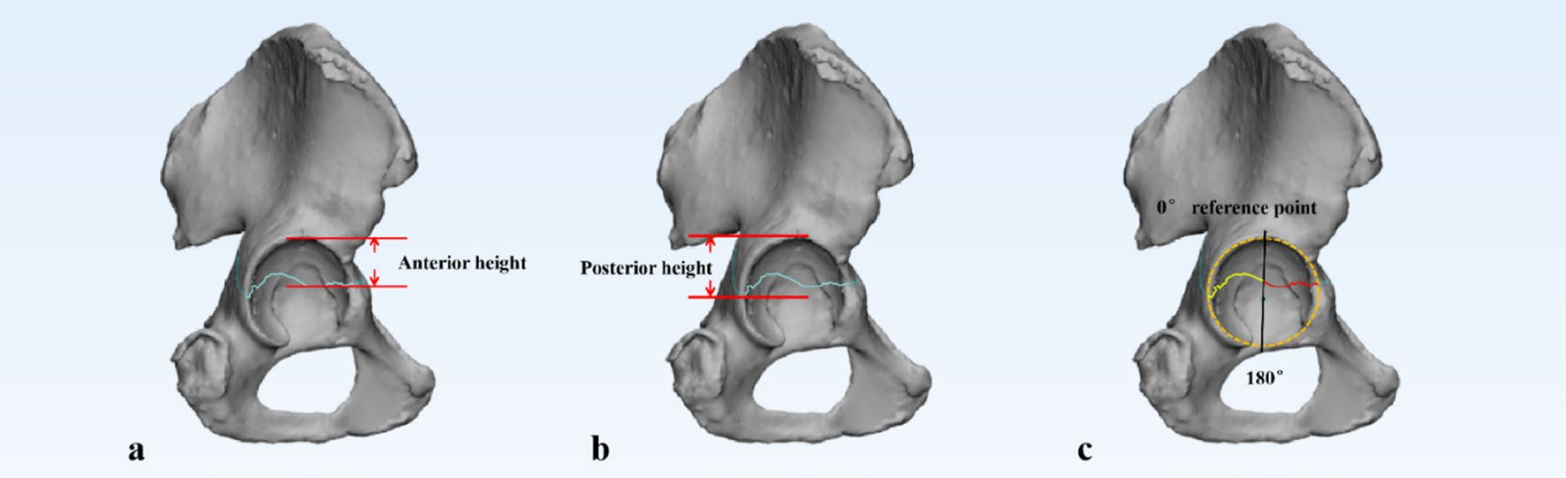
**a**, **b** Anterior height or posterior height of the transverse fracture: the distance from the anterior or posterior rim fracture position to the tangent line at the top of the acetabular joint on a lateral three-dimensional reconstruction image, respectively. **c** The acetabular surface was divided into anterior and posterior halves by a line extending between the 0° to 180° reference points. The length of the anterior or posterior intra-articular fracture line is marked in red and yellow, respectively. The length of the fracture lines could be measured in the 3D view by using 3-Matic software

To quantitatively analyze the fracture characteristics, we defined some new anatomic parameters. As mentioned in a previous acetabular fracture mapping report, the articular surface can be represented as a clock face [18]. During CT scanning from the cranial end to the podalic end of the body, the tangent point at the top of the acetabular joint is regarded as the 0° reference point (Fig. 2a, b). The anterior rim fracture angle was defined as the intersection between the 0° reference point and the fracture position at the anterior rim. The posterior rim fracture angle was measured between the 0° reference point and the fracture position at the posterior rim using the same method (Fig. 2c, e). The anterior or posterior height [17] of the transverse fracture was also measured on the lateral view of the 3D reconstruction image (Fig. 3a, b). The lengths of the intra-articular fracture lines in the anterior or posterior regions were obtained in 3-Matic software (Fig. 3c).

### Statistical analysis

Both quantitative and descriptive analyses of fracture mapping were conducted. Statistical analysis was performed with SPSS 25.0 software (IBM, Armonk, NY, USA). Descriptive statistics were used to analyze the patient data, which are presented as the mean ± standard deviation. One-way analysis of variance (ANOVA) and Scheffe’s post hoc tests were used to compare continuous data among the three fracture patterns. The lengths of the anterior or posterior intra-articular fracture lines were compared using independent t tests. A value of *P* < 0.05 was regarded as significantly different. Fracture morphological mapping was analyzed through descriptive statistics.

## Result

The demographic information and characteristics of the patients are summarized in Table 1. Forty-nine transverse acetabular fractures were identified in 49 patients and included in this study. Among these patients, there were 32 males and 17 females, with a mean age of 42 years (range 21–74 years). Left-sided fractures were found in 27 adults, and right-sided fractures were found in 22 adults. Concomitant disruption of the pelvic ring was observed in 33 cases. Motor vehicle collisions were the most common injury mechanism.

**Table 1:** Demographic information of patients

Variable	Patients (*n* = 49)
Mean age, year (SD)	42 (13)
*Sex, n (%)*	
Male	32(65.3)
Female	17(34.7)
*Side of injury, n (%)*	
Right	22(44.9)
Left	27(55.1)
*Concomitant disruption of the pelvic ring, n (%)*	
Yes	33(67.3)
No	16(32.7)
*OTA/AO classification, n (%)*	
62.B1.1	19 (38.8%)
62.B1.2	17 (34.7%)
62.B1.3	13 (26.5%)
*Injury mechanism, n (%)*	
Motor vehicle collision	32 (65.3%)
Fall from height	8 (16.3%)
Others	9 (18.4%)

*SD* standard deviation, *AO/OTA* AO Foundation/Orthopedic Trauma Association

Each case was further classified based on the AO/OTA classification. The measurements of the anatomic parameters in each pattern are summarized in Tables 2 and 3.

**Table 2:** Comparison of anatomic parameters in three subgroups

Variable	Type 62-B1.1 (*n* = 19)	Type 62-B1.2 (*n* = 17)	Type 62-B1.3 (*n* = 13)	*P*
Anterior rim fracture angle (°)	74.5 ± 9.4	68.5 ± 5.9	65.4 ± 17.3	0.071
Posterior rim fracture angle (°)	107.5 ± 25.0	98.3 ± 17.7	62.7 ± 23.2	0.000
Anterior height (cm)	2.1 ± 0.4	1.8 ± 0.3	1.7 ± 0.7	0.072
Posterior height (cm)	3.5 ± 1.0	3.2 ± 0.7	1.7 ± 0.9	0.000

**Table 3:** Comparison of fracture line length

Variable	Anterior intra-articular fracture line length (cm)	Posterior intra-articular fracture line length (cm)	*P*
Type 62-B1.1 (*n* = 19)	4.1 ± 0.2	4.5 ± 0.4	0.001
Type 62-B1.2 (*n* = 17)	3.7 ± 0.2	4.2 ± 0.4	0.000
Type 62-B1.3 (*n* = 13)	3.6 ± 0.5	3.9 ± 0.6	0.280

### Overall distribution

**Fig. 4:**
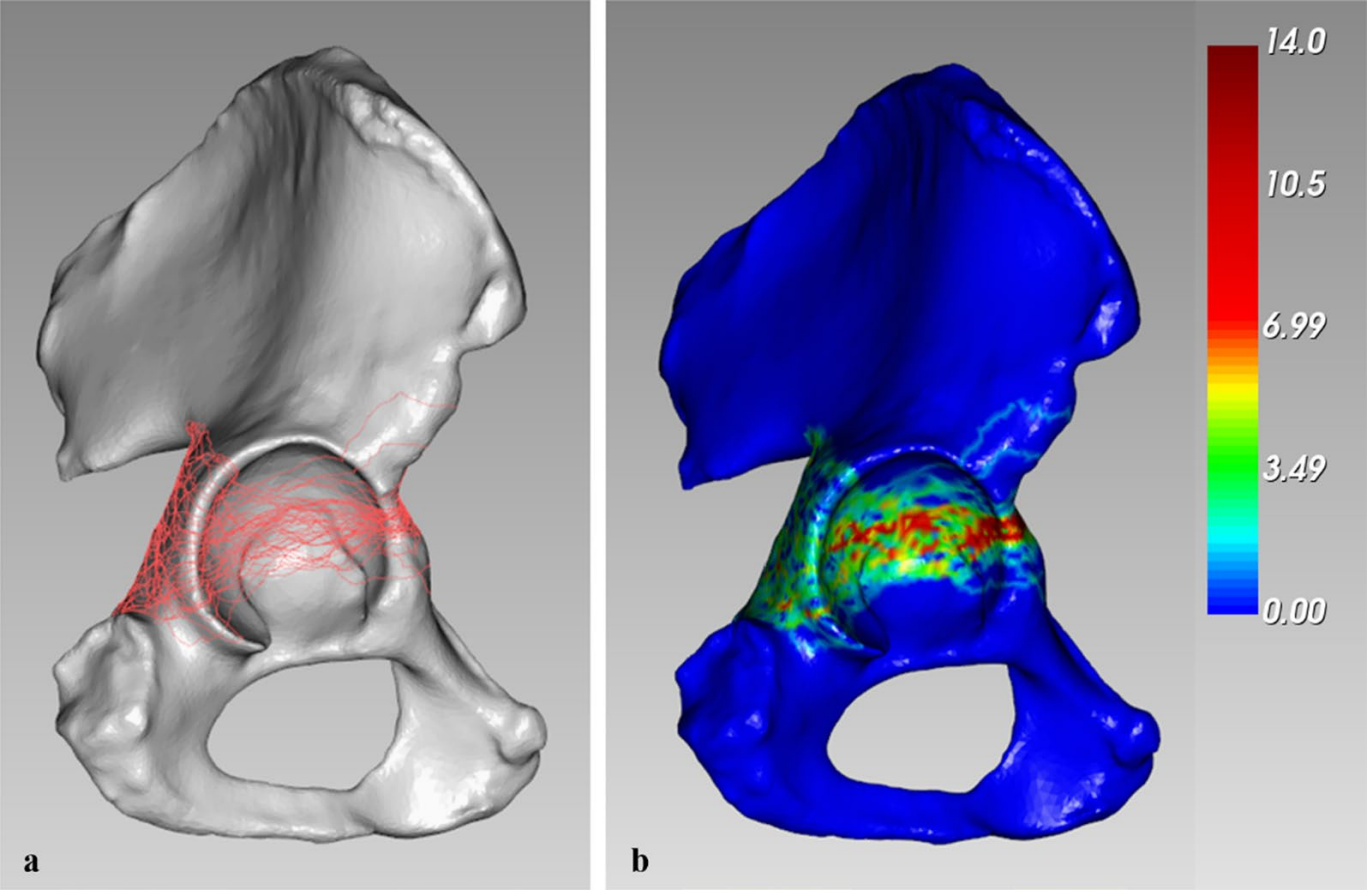
Fracture map and heatmap of 49 superimposed transverse acetabular fractures. All of the fracture lines were transferred into E-3D. Fracture lines are shown in red on the right-sided standard model. The heatmap illustrates the frequency of fracture locations using a color scale bar. Red represents a higher fracture line density. **a** Fracture line mapping of the articular surface. **b** 3D heatmap

The fracture lines of 49 cases were drawn, and the overall distribution of transverse acetabular fractures was visualized through fracture mapping (Fig. 4a). The average anterior rim fracture angle was 70.0° (± 11.6°), and the posterior rim fracture angle was 92.4° (± 28.5°). Moreover, anterior rim fracture angles in 40 cases (40/49, 81.6%) fell within a wide range between 63° and 80°. Posterior rim fracture angles in 35 cases (35/49, 71.4%) were determined to be in the 84°–127° range.

On the heatmap (Fig. 4b), the hot zones were located around the highest position of the cotyloid fossa and the narrowed region. The cold zone was located on the inferior third of the articular surface.

### AO/OTA 62-B1.1 (Infratectal pattern)

**Fig. 5:**
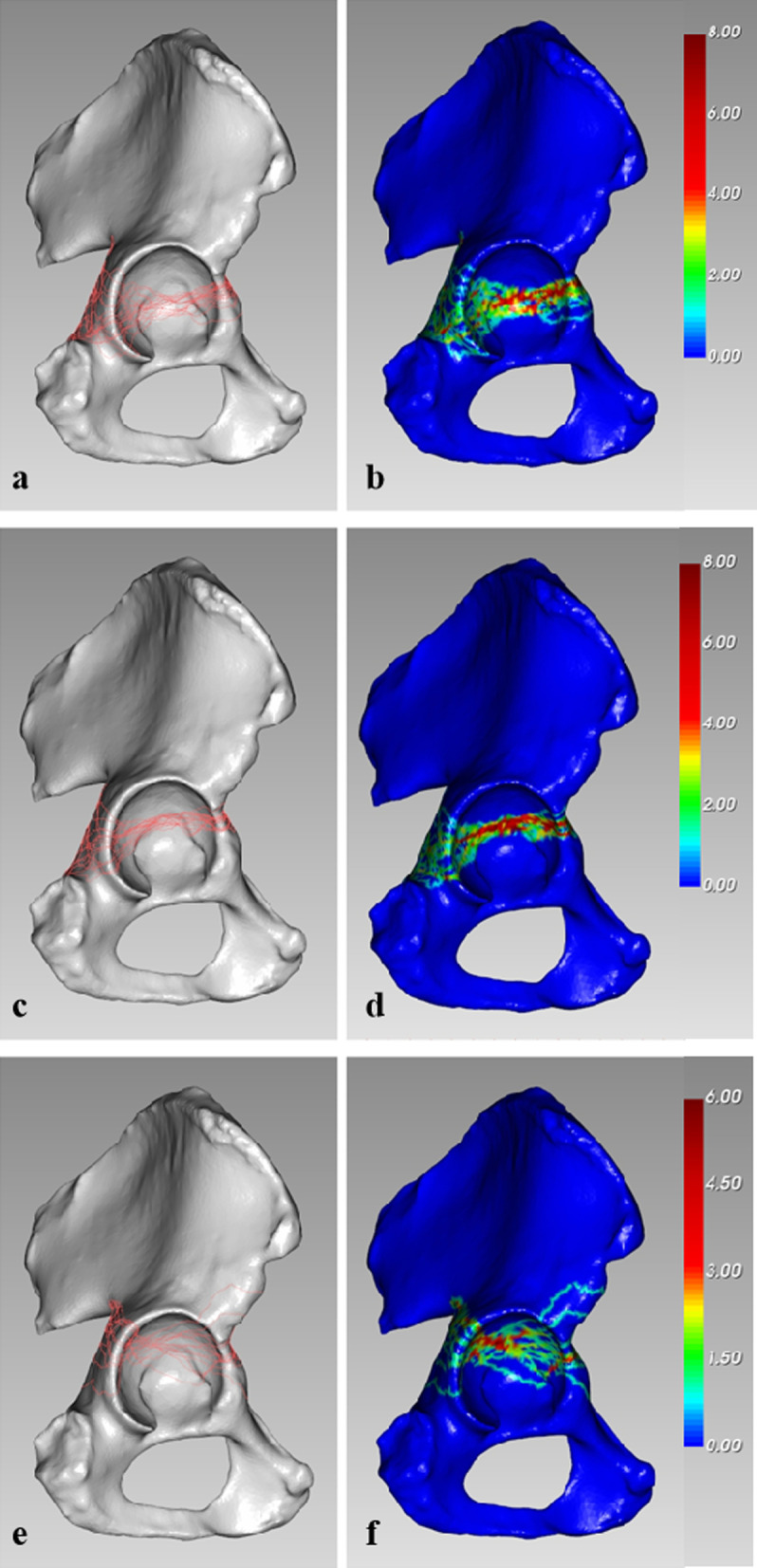
Different fracture and heatmap results based on the AO/OTA classification: **a**, **b** type 62-B1.1 (infratectal pattern); **c**, **d** type 62-B1.2 (juxtatectal pattern); and **e**, **f** type 62-B1.3 AO/OTA 62-B1.3 (transtectal pattern)

There were 19 fractures (19/49, 38.8%) of this type, and the anterior and posterior walls of the lower acetabulum were separated. The fracture lines in the posterior area were radially distributed and highly scattered. The anterior fracture lines were concentrated on the narrowed zone, which was the junction of the ilium and ischium (Fig. 5a, b).

### AO/OTA 62-B1.2 (Juxtatectal pattern)

There were 17 fracture cases (17/49, 34.7%) in this group. The fracture lines of this pattern most commonly occurred on the middle part of the articular surface and were distributed horizontally. The main fracture lines passed through the highest point of the cotyloid fossa in the region where the roof is internally limited. The anterior fracture lines were approximately superposed. The distribution of posterior fracture lines was similar to that for the infratectal pattern but less widespread. However, the fracture lines scarcely involved the acetabular dome (Fig. 5c, d).

### AO/OTA 62-B1.3 (Transtectal pattern)

There were 13 fractures (13/49, 26.5%) passing at the level of the roof that were included in this pattern. The site of the fracture lines was distributed quite variably. Fracture lines were distributed diffusely and irregularly emerged toward both the anterior and posterior regions. The recurrent fracture lines mainly converged on the posterior region of the acetabular dome (Fig. 5e, f).

### Anatomic parameter analysis

The anterior rim fracture angle and the anterior height were comparable among all patterns (*P* = 0.071, *P* = 0.072). Post hoc tests of the posterior rim fracture angle and the posterior height showed that all three patterns were significantly different from one another when compared pairwise (*P* < 0.01; Table 2). The posterior intra-articular fracture line was significantly longer than the anterior intra-articular fracture line in type 62-B1.1 and type 62-B1.2 fractures (*P* < 0.01). However, the lengths of the anterior and posterior intra-articular fracture lines in type 62-B1.3 fractures were not significantly different (*P* = 0.280; Table 3).

## Discussion

In the present study, the 3D fracture and heatmapping techniques were applied to offer a comprehensive morphological demonstration of transverse acetabular fractures. We also combined qualitative and quantitative outcomes to summarize the distribution and frequency of fracture lines in transverse fractures. This study found that the distribution of the fracture lines on the anterior acetabular rim was related to the anatomical features of the narrowed zone, and the fracture lines on the posterior rim were radially distributed. Intra-articular fracture lines were distributed on the superior and middle thirds of the joint. The recurrent fracture lines of the acetabular dome mainly converged on the posterosuperior articular surface.

The innominate bone is formed as a coalescence of three parts (ilium, pubis, and ischium) before the age of eighteen [19]. Prior to this, the three bones are combined by a cartilaginous T-shaped structure called “triradiate cartilage,” which is regarded as the acetabular weakness zone [20]. The isthmus or narrowed region located at the junction of the ilium and pubis has been described as the elective site of transverse fractures. Theoretically, the isthmus exists, resulting in observable transverse fracture lines at this location. Our findings support that common anterior fracture lines are highly likely to travel through the narrowed zone. The comparison of anatomic parameters among the three fracture patterns also showed that anterior recurrent fracture lines were concentrated mainly on a relatively consistent location, but the posterior fracture line distribution was diffuse (Table 2). Based on the mapping of 49 transverse acetabular fracture cases, the narrowed zone of the anterior column was identified as the recurrent fracture region, which is approximately in the 63°–80° range, where 81.6% of fracture lines occur. Hence, from a morphological point of view, it can be determined that the special anatomical architecture of the narrowed region contributes to the anterior fracture lines converging this position.

In this study, the mapping results demonstrated that the height of the transverse fracture line and its obliquity are ever-changing. In contrast to the above-mentioned analysis of anterior recurrent fracture lines, the posterior fracture line distribution is highly scattered and resembles an irregular radial pattern. According to Herman et al. [2], each displacement vector represents a unique hallmark feature of the fracture line. In other words, the fracture line location is dependent on the injury mechanism, including the load of the impact, the direction of the injurious force, and the precise position of the femoral head at the moment of injury. Dakin et al. [21] showed in their study that acetabular fracture patterns associated with motor vehicle crashes correlated with the type of impact and verified the biomechanical concepts proposed by Herman et al. Although previous cadaveric specimen studies and finite element analysis have accelerated the biomechanical analysis of acetabular fractures, reproducible acetabular fracture models with consistent fracture line locations are still difficult to create in the laboratory [22, 23]. Further mapping studies could be combined with biomechanical experiments to explore the definite mechanism of fracture.

Simple transverse fractures are traditionally regarded as transverse-oriented acetabular fractures according to the common concomitant disruption of the pelvic ring [5, 24, 25]. However, there is no clear theorem to confirm the impact direction during the fracture. Based on the mapping results, intra-articular fracture lines were mainly located on the superior and middle thirds of the joint surface. This may be a good explanation for why transverse fractures have been classified into a superomedial displacement vector pattern [2]. As the heatmap showed, the hot zones were coincidently concentrated at the highest position of the cotyloid fossa and the narrowed region. Thus, the mid-posterior recurrent fracture region of the acetabulum might be due to the force acting between this structure and the femoral head. In contrast, fractures in the narrowed zone might result from the weaker structure of this region, instead of from direct impact. This means that the direction of the impact from the femoral head might be toward the mid-posterior portion of the superior acetabulum. The correlation between our mapping study and the present injury mechanism analysis may help to improve clinical effect in the treatment of transverse fractures.

The AO/OTA 62-B1 classification, which is based on Letournel’s theory, represents transverse acetabular fractures and further subdivides them according to the level at which the fracture breaks the acetabulum and whether the ventrocranial area was involved [26]. Our mapping study illustrates the variations in fracture lines among subtypes. For the transtectal pattern (AO/OTA 62-B1.3), all fractures transgressed the weight-bearing dome, and the majority of the recurrent fracture zone lines emerged on the posterosuperior articular surface (Fig. 5e, f). Because reduction of the weight-bearing dome must be perfect [27], a posterior approach (e.g., the Kocher–Langenbeck approach) may be the preferred option for anatomical reduction under direct vision for this pattern. For the other two groups (AO/OTA 62-B1.1 and AO/OTA 62-B1.2), we found that the fracture lines seldom involved the weight-bearing surface (Fig. 5a–d). However, in these two subtypes, the anterior and posterior walls were usually separated in the lower region. Based on the obtained measurements, the posterior intra-articular fracture line was significantly longer than the anterior intra-articular fracture line in these two subgroups. This finding demonstrated that the fracture severity was higher in the posterior hemisphere than in the anterior hemisphere. Because the acetabular posterior wall plays a crucial role in maintaining the stability of the hip joint, high-quality reduction of the posterior walls can result in satisfactory clinical outcomes [28, 29]. The Kocher–Langenbeck (KL) approach may be appropriate in these situations to easily restore posterior fractures. In brief, our study recommends that transverse acetabular fractures have a high probability of being successfully reconstructed through the KL approach. However, surgical treatment protocols are generally affected by many factors, such as the surgeon’s experience and the fracture line locations [27, 30]. The optimal approach for open reduction and internal fixation of transverse fractures remained controversial in a previous study [31, 32]. In general, orthopedic surgeons prefer fractures that can be appropriately addressed with a single approach (either an anterior or posterior approach), while the exposed column is directly reduced and the other column is indirectly reduced [33]. However, one issue for less experienced surgeons is how to correctly decide on the sequence of events in open reduction and whether simultaneous anterior and posterior approaches might be essential. This study can be used as a reference for selecting surgical approaches during the formulation of a surgical strategy.

Several limitations in this retrospective study should be acknowledged. First, the sample size of this study was small. Some patients with no or insufficient CT scans were not included, which might have limited the generalizability of the study results. Second, owing to the great anatomical variability and size differences of the hemipelvis in different patients, system errors were inevitable during manual manipulation in fracture mapping. Third, the direction and distance of the displaced fracture fragments were not explored in this study due to the occurrence of concomitant pelvic ring disruptions, and transverse acetabular fractures were common in many cases, which resulted in a lack of accurate references for affected innominate bone reduction to the contralateral side.

In conclusion, transverse acetabular fractures occurred more frequently in the anterior narrowed zone, and the fracture lines located on the posterior were diffusely distributed. The intra-articular fracture line distribution mainly concentrated on the superior and middle thirds of the joint surface. The recurrent fracture lines involving the weight-bearing surface were mostly centered on the posterior of the acetabular dome. This study will not only be useful in guiding the selection of surgical approaches during the formulation of a surgical strategy but can also provide a visual framework for surgeons to understand the characteristics of transverse fractures.

## Data Availability

All the data were contained in the article.

## Abbreviations

CT: Computed tomography
3D: Three-dimensional
AO/OTA: AO Foundation/Orthopedic Trauma Association
